# Understanding Oriental Medicine Using a Systems Approach

**DOI:** 10.1093/ecam/nep037

**Published:** 2011-06-22

**Authors:** Jong Yeol Kim, Duong Duc Pham

**Affiliations:** ^1^Department of Medical Research, Korea Institute of Oriental Medicine, Daejeon 305-811, Republic of Korea; ^2^Department of Internal Medicine, National Hospital of Traditional Medicine of Vietnam, Hanoi, Vietnam

## Abstract

Two international meetings, the International Physiome Symposium 2008 and the Workshop on Systems Biology (SB) and Oriental Medicine (OM), were held to discuss the most appropriate scientific tools to research OM. Participants agreed that since OM is holistic medicine it needs a systems approach such as SB. However, SB itself is still a long way from identifying the high-level organization processes in the biological system that might correlate with concepts in OM. As such, a modest goal of launching a project to examine the problems of translation and interpretation of OM concepts would be the first step.

## 1. Introduction

Oriental medicine (OM), which is defined by World Health Organization as East Asian medicine, is a theory-based medicine that originated from ancient China and it was speedily absorbed, modified and practiced within several China's traditional cultural influenced countries (Korea, Japan and Vietnam) [1]. Unlike Western medicine (WM) constituted from various outcomes of experimental research designs coming along with the development of a great number of disciplines, OM is based on systematic observations and knowledge inherited from generation to generation. The concepts and theories of OM were postulated to give convincible explanations to physio-pathological phenomena. Concepts such as Qi (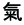), Yin (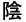), Yang (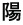) and so on are unfamiliar to WM doctors whose background is scientific trust. During several last decades, OM is transplanted and developed rapidly in the West that may be due to its real effective curativeness, despite its insufficient scientific evidence and vague concepts. Scientists have been fascinated to evaluating OM; however, most current researchers simply assess OM in biomedical terms by reductionistic approach. This trend could only draw separate pieces of a picture, but it is unable to understand OM as the whole [2].

Along with the development of science, researchers think that they may need more appropriate tools to tap into the deeper secrets of OM. Two international meetings were recently held to discuss the most appropriate scientific tools to research OM. The International Physiome Symposium 2008 was held in Seoul, Korea, on April 10-11, 2008, organized by Korea Institute of Oriental Medicine and the Korean Physiome Society. The Workshop on Systems Biology (SB) and Oriental Medicine was held at Oxford University, UK, on July 9, 2008, organized by Prof. Denis Noble and Dr Jong Yeol Kim. This article aims to highlight and discuss on the main themes of these meetings.

## 2. Toward Holistic Medicine: A Need of System Approach

There have been many descriptions of OM so far, but some remarkable ideas were proposed in these meetings. Prof. Denis Noble (University Laboratory of Physiology, Oxford University, UK) described OM as holistic medicine characterized by a system of multi-action medications, especially in the case of herbal remedies. Elements of OM such as Qi (Ki, 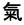), Yin (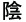) and Yang (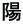) are difficult to define, and refer to high-level states that do not map to elements of reductive biology, which provides low-level data. As such, OM is usually rejected and neglected by most reductionists. The neglect and rejection of OM in countries where WM is dominant is mostly due to historical/cultural barriers. In the West, many people believe that OM includes “magic”, and that it is somewhat shamanistic. Prof. Yung E Earm (Seoul National University) commented that the characteristic of human nature could be described as the balance between body and mind, and the status of balance between antagonist organs and systems. These concepts are similar to those of the *milieu interior* proposed by Claude Bernard (1865) or homeostasis proposed by Walter Canon (1932) [3]. Prof. ZhiDao Xia (Oxford University) highlighted the application of Traditional Chinese Cosmology, which understands the universe as a whole, in the fundamental theory of OM. He drew attention to some interesting numerological comparisons between ancient Chinese cosmology and modern Western analysis, and indicated how the characteristics and movements of Yin-Yang correspond with the Big Bang model, and binary and genetic codes, and how the five elements correspond with the ecosystem. Dr Jong Yeol Kim (Korea Institute of Oriental Medicine) presented recent achievements in scientific studies on the Sasang Constitutional Medicine (SCM), the unique medical theory of Korean Traditional Medicine named as the Four Constitution Medicine by WHO, invented over 100 years ago by Dr Je Ma Lee [2, 3]. SCM classifies human beings into four constitutions which differ in terms of (i) sensitivity to certain groups of herbs and medicines, (ii) equilibrium among internal organic functions and (iii) physical and psychological characteristics. SCM emphasizes the mutual relationship between the mind and the body, and therefore diseases occur due to the abnormal interaction of these two parts [4].

Since OM understands humans and health as a whole, conventional research approach is insufficient to give an insight into the essence of OM and as such specific systems tools are required.

## 3. SM and OM: Could There Be a Synthesis?

SB, a biology-based interdisciplinary study, focuses on complex interactions of multiple genes, proteins, transmitters, hormones and drugs. By measuring many genes, proteins and metabolite at the same time, SB may provide a comprehensive view of human functional process and its entire response to a disease or a therapeutic stimulation. This source of data provides foundation to promote a trend of new approach within the concept “physiome”. The term “physiome” derives from the Latin “*physio*” (life) and “*ome*” (as a whole). In that sense, “physiome” is a term denoting the view of human life in complex state.

Prof. Denis Noble stated that biological functionality is multi-leveled, and that the transmission of information in the body is not one way. Moreover, DNA is not the sole transmitter of inheritance, and that epigenetic marking can also be transmitted via sperm. The self is an integrative process not an object or substance, thus a multi-level analysis is necessary. There are some questions that may be considered. (i) Since SB, known as the “middle-out approach”, is more holistic than reductive biology, and OM is also holistic, could “downward causation” be a proper way to link SB and OM [5]? (ii) Since SB identifies multiple actions rather than single genes or proteins, could SB provide a means of understanding and developing herbal medicine, which is based on the synergistic actions of multiple components? (iii) Since SB recognizes the importance of control of the genome by high levels including behavioral and social factors via epigenetic marking and control, could it open the dialogue on the central role of the mind in OM?

In a line with this viewpoint, Prof. Leroy Hood postulated in his recent publication that biological information consists of digital information of the genome and the environmental signals arising outside the genome. These two types of information integrate in a state of comprehensive mutual interaction that influences organisms in their development and responses to environments. The genome contains two major types of information, genes and control elements, which interact each other to form network structures of a system. This network may be perturbed simultaneously or by pathological environmental cues, such as infectious agents or chemical carcinogens, leading to a manifestation of disease state. Since SB qualifies all of the molecular elements of a biological system to assess their interactions and to integrate that information into graphical networks model, SB would be an appropriate tool to understand the human disease-perturbed networks, a requirement to provide a more specific therapy, prediction and prevention for a particular disease occurred to specific individuals. These outcomes will initiate a new and effective medicine, a personalized medicine [6].

The term “*perturbed networks*” of Prof. Leroy Hood is a reminiscent of the concept of “*disharmony*” (
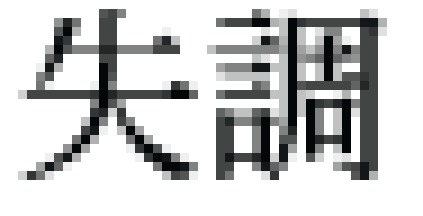
) in OM, which means an imbalance and incoordination. According to OM, diseases and ailments are occurred when there is an appearance of “disharmony” among inner functions and/or between inner functions and external environment. Moreover, individuals are different in constitutional and healthy states. As such, health problem needs a holistic and individualized curativeness and a system approach such as SB should be used to interpret OM.

Prof. Yung E Earm noted the difficulties in matching the body organ/system and the functional regulatory system between WM and OM. A great challenge is to translate terms into more general concepts that can be understood in both disciplines. He presented a project to provide basic physiological and pharmacological characteristics of human population groups. That project, using a middle–out approach, aims to determine what kind of physiological parameters are responsible for different constitutions or characters at the system, organ and cellular levels. Key parameters would cover a variety of body functions, systems and characteristics, such as pulmonary, kidney, digestive, liver, autonomic nervous system, heart, circulation, blood type, skin, voice and psychological factors. Based on those data, a human constitution simulation model can be constructed, which can then lead to the ultimate goal of a virtual human constitution model. Recently, a project on the Virtual Physiological Human was launched under the financial support of the European Commission. This project aims to develop a methodological and technological framework to facilitate investigation of the human body as an integrated system, which concerns a variety of scientific disciplines (biology, physiology, biophysics, biochemistry, molecular biology and bioengineering), dimension scales (body, organ, tissue, cells and molecules) and anatomical sub-systems (cardiovascular, musculoskeletal and gastrointestinal) [7].

## 4. Controversy Surrounding SB

Despite a general consensus that SB could be used as a system tool to understand OM, there were some dissenting opinions. Dr Shanshan Wang (University of Cambridge) criticized that: (i) SB is still a relatively new field and is not sufficiently mature to be used as a tool to study OM; (ii) computer modeling is in no way as reliable as animal experiments; (iii) Chinese research coupled with poor funding is insufficient to perform such cutting-edge studies.

Dr Eric Werner (Oxford University) appeared to be more practical with his comment that SB does open the way to a more constructive dialogue, but that SB itself is still a long way from identifying the high-level organization processes in the biological system that might correlate with concepts in OM. He suggested it was important to identify modest goals.

Prof. Denis Noble agreed that while SB is young, it has undergone a long development since Claude Bernard first presented his ideas in 1895 [8]. He acknowledged two questions needed to be addressed: is SB sufficiently mature to be applied, and how can it be developed to become a credible tool to research OM? He concluded that the first step of the project should be focused on more modest aims dealing with the problem of translation and interpretation of OM concepts.

## 5. Problems of Translation and Interpretation

Many efforts were implemented to translate OM concepts to scientific terms but no equivalent terminology was accepted so far. Prof. Kan Wen Ma (London University) stated that while OM refers to five viscera—heart, lung, liver, spleen and kidney—it does not define these organs with the same anatomical boundaries as WM. These viscera are considered to functions more like systems than organ units. Another example is the concept of “Mai” (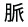), which is understood as the conduit through which Qi and blood pass, was commonly translated as “meridians”, but the other translation as “channels and collaterals” might be more accurate and clearer. Dr Jong Yeol Kim used the organ concepts of SCM as an example of translation problems, in that the five viscera are also considered as systems rather than organs as in WM. For example, in SCM the lung may refer to the whole respiratory system, while the spleen refers to the whole digestive system [3]. Dr Eric Werner also pointed out that while concepts in OM and oriental ontology (OO) may be superficially translated into WM and Western ontology (WO), those Western translations do not provide sufficient richness to explain the actual practice of OM. He believes that to understand OM in WM, we must also translate the ontology and methodologies of practice, and that relating OM pragmatic meaning and practice to the curative effects of OM is the key problem in understanding OM in terms of WM.

Prof. Denis Noble commented that a “translation” may not be the best way of characterizing what is required since the word, or character itself, does not always function in the same way within the lexical and semantic frame of different languages. He quoted works by Elisabeth Hsu (2001) that Qi (Ki) is sometimes used as a categorizer rather than a noun with meaning in its character [9]. Thus, it may be better to use the word ‘interpretation' rather than ‘translation'. He used the example of translation in poetry, where insistence on accuracy of wording can destroy the poetic insight. Similarly, not everything in OM can be mapped simply in a Western language. Therefore, accuracy does not mean one-to-one mapping.

## 6. Future Orientation

As a way of reaching the ultimate target, the process should begin with focusing on the modest goal of examining the problems of translation and interpretation of OM concepts. A preliminary step is to initiate interaction between OM and the virtual physiological human. It is promising that this initiative is going to be promoted in the framework of the International Human Physiome Project.

## Funding

Korea Science and Engineering Foundation (KOSEF); Korea Ministry of Education, Science and Technology (MEST) (under the project no. M10643020004-08N4302-00400).
